# Thoughts on ‘Efficacy of RestoreX after prostatectomy: open‐label phase of an RCT’

**DOI:** 10.1002/bco2.320

**Published:** 2024-02-22

**Authors:** Mustafa Ganijee, Rasi Mizori, Awab Ahmad, Mirza Hashim Ahmad, Malik Takreem Ahmad

**Affiliations:** ^1^ Ashton GP Service Ashton‐under‐Lyne UK; ^2^ GKT School of Medical Education King's College London London UK; ^3^ St George's University of London London UK; ^4^ Kent and Medway Medical School Canterbury UK

To the Editor,

We congratulate Zganjar et al. on their evaluation of the effects of RestoreX penile traction therapy following post‐prostatectomy.^1^ This single‐centre, randomised trial offers valuable insights into the impact of RestoreX on a common yet under‐researched complication: reduced penile length. Given that prostate cancer is the most common cancer worldwide,^2^ and prostatectomy remains the definitive treatment of choice, this study addresses some important concerns related to post‐prostatectomy complications and potential management strategies.

However, several factors might constrain the generalisability of these findings. Firstly, the study's narrow demographic scope and its single‐centre design mean that lack of patient diversity is a limitation. With the inclusion of 82 men, only 45 of whom there is 9‐month data available for, extrapolating these results to the broader population is challenging. A single‐centre study inherently reflects the patients within that specific centre and does not fully reflect the diverse demographics of patients affected by this complication globally. Post‐prostatectomy complications are a pervasive issue, affecting a myriad of patient demographics worldwide,^3^ and so this underscores the importance of research encompassing a broader range of demographics.

We also noted the significant dropout rate given the study's limited participant pool. With 45 participants out of an initial 82, this indicates a dropout rate of more than 45% of participants, further restricting the data's generalisability. Such significant dropout rates can introduce biases, and it is therefore essential to understand the reasons for these dropouts, as these may reveal potential challenges associated with RestoreX. The small sample size inherently diminishes the study's power, and this impacts the interpretation and subsequent conclusions.^4^

Furthermore, the study monitors outcomes up to 9 months for the 45 men who completed the whole study. Although these findings are promising, the ramifications of a prostatectomy and the trajectory of post‐operative recovery can span years.^5^ A longer follow‐up would offer deeper insights into RestoreX's long‐term effects and reveal any latent side effects that might not manifest during shorter usage periods. Such comprehensive data would allow both patients and healthcare providers to have a holistic understanding of the medications, encompassing their prolonged effects and potential side effects.

To conclude, while Zganjar et al. study sheds light on the benefits of RestoreX treatment on penile length decrease, further studies should focus on including patients from a broader demographic to represent the diverse populations best that this complication affects. We urge further exploration into the reasons behind more than 45% of participants in the study dropping out and how this cause can be managed to increase adherence, particularly in the real world. Finally, we hope that future studies will explore the effects of RestoreX for longer periods of time, thereby offering insight into its long‐term effects and any associated complications/side effects that patients and healthcare practitioners should be aware of.

Sincerely,

Dr Mustafa Ganijee

General Practitioner

Ashton GP Service, 193 Old Street, Aston‐under‐Lyne, OL6 7SR

## CONFLICT OF INTEREST STATEMENT

I, Dr Mustafa Ganijee, the first author, wish to declare that I am a general practitioner with a specialist interest in male sexual health, especially with regard to the private treatment of erectile dysfunction and premature ejaculation. Thus, the opinions and critical commentary contained within this letter are informed by my clinical experience. This personal interest does not, however, influence the integrity or objectivity of my professional perspective. The points presented below are presented on the basis of scientific evidence and unbiased professional judgement. There are also no conflicts of interest or financial interests that have directly or indirectly influenced this letter to the editor.
